# A systematic review of microbial markers for risk prediction of colorectal neoplasia

**DOI:** 10.1038/s41416-022-01740-7

**Published:** 2022-03-15

**Authors:** Lili Yu, Gang Zhao, Lijuan Wang, Xuan Zhou, Jing Sun, Xinxuan Li, Yingshuang Zhu, Yazhou He, Kleovoulos Kofonikolas, Debby Bogaert, Malcolm Dunlop, Yimin Zhu, Evropi Theodoratou, Xue Li

**Affiliations:** 1 Department of Big Data in Health Science School of Public Health, Center of Clinical Big Data and Analytics of The Second Affiliated Hospital, Zhejiang University School of Medicine, Hangzhou, China; 2 Center for Disease Control and Prevention of Hangzhou, Hangzhou, China; 3 Department of Colorectal Surgery and Oncology, Key Laboratory of Cancer Prevention and Intervention, Ministry of Education, The Second Affiliated Hospital, Zhejiang University, Hangzhou, China; 4 Department of Oncology, West China School of Public Health and West China Fourth Hospital, Sichuan University, Sichuan, China; 5 Faculty of Medicine, University of Thessaly, Volos, Greece; 6 Centre for Inflammation Research, University of Edinburgh, Edinburgh, UK; 7 Colon Cancer Genetics Group, Institute of Genetics and Molecular Medicine, University of Edinburgh, Edinburgh, UK; 8 Centre for Global Health, Usher Institute, University of Edinburgh, Edinburgh, UK; 9 Cancer Research UK Edinburgh Centre, Institute of Genetics and Cancer, University of Edinburgh, Edinburgh, UK

**Keywords:** Prognostic markers, Microbiome

## Abstract

**Background:**

Substantial evidence indicates that dysbiosis of the gut microbial community is associated with colorectal neoplasia. This review aims to systematically summarise the microbial markers associated with colorectal neoplasia and to assess their predictive performance.

**Methods:**

A comprehensive literature search of MEDLINE and EMBASE databases was performed to identify eligible studies. Observational studies exploring the associations between microbial biomarkers and colorectal neoplasia were included. We also included prediction studies that constructed models using microbial markers to predict CRC and adenomas. Risk of bias for included observational and prediction studies was assessed.

**Results:**

Forty-five studies were included to assess the associations between microbial markers and colorectal neoplasia. Nine faecal microbiotas (i.e., *Fusobacterium, Enterococcus, Porphyromonas, Salmonella, Pseudomonas, Peptostreptococcus*, *Actinomyces, Bifidobacterium* and *Roseburia*), two oral pathogens (i.e., *Treponema denticola* and *Prevotella intermedia*) and serum antibody levels response to *Streptococcus gallolyticus subspecies gallolyticus* were found to be consistently associated with colorectal neoplasia. Thirty studies reported prediction models using microbial markers, and 83.3% of these models had acceptable-to-good discrimination (AUROC > 0.75). The results of predictive performance were promising, but most of the studies were limited to small number of cases (range: 9–485 cases) and lack of independent external validation (76.7%).

**Conclusions:**

This review provides insight into the evidence supporting the association between different types of microbial species and their predictive value for colorectal neoplasia. Prediction models developed from case-control studies require further external validation in high-quality prospective studies. Further studies should assess the feasibility and impact of incorporating microbial biomarkers in CRC screening programme.

## Introduction

Colorectal cancer (CRC) is the world’s third most common cancer and the second leading cause of cancer death [1]. It is reported that men have a higher risk of developing CRC compared to women, and women have up to 25% lower risk of CRC mortality than men [2]. Previous evidence suggests that this sex-specific difference could be attributed to the differential exposure to sex hormones, especially to oestrogen [3]. Elevated nuclear oestrogen receptor beta expression is independently associated with a better overall survival in female patients, revealing that the oestrogen receptor beta may be involved in underlying mechanisms in CRC [4].

Although substantial research has been conducted, a full understanding of the complex aetiology of CRC remains elusive, as well as the pathogenesis of progression. Increasing evidence is revealing that dysbiosis of the gut microbiome may be involved in the pathogenesis of CRC, which may lead to chronic metabolic and inflammatory changes and thus promote colorectal carcinogenesis [5–7]. For example, exposure to common prescription drugs (e.g., proton pump inhibitors and oral antibiotics) might influence the dysbiosis of gut microbiome and therefore contribute to the development of neoplastic lesions [8]. Apart from their potential for carcinogenesis, associations between gut bacteria and clinical outcomes of CRC have raised the possibility of using them as prognostic markers. Several molecular epidemiology studies have reported an inverse association between the tumour-associated *Fusobacterium nucleatum* and CRC survival [9, 10]. In addition, the gut microbiota may modulate the response to cancer therapy and susceptibility to toxic adverse effects, thereby affecting outcome, although there is only limited evidence for this [11, 12].

In recent years, many countries have introduced organized screening programme to increase early CRC detection followed by colonoscopy if needed [13]. Importantly, there is evidence that changes in the gut microbiome may occur during the early stages of colorectal carcinogenesis and can be used to identify individuals at risk. Changes in the microbiome over time might therefore be used as biomarkers for the early detection of colorectal neoplasia, and for improving screening strategies [14]. The interest is further encouraged by the fact that bacterial DNA can be successfully isolated from quantitative faecal immunochemical test (qFIT) cartridges [15] and used for risk prediction/stratification complementing existing qFIT screening programme. Microbial markers could be used as a complementary test for qFIT, especially among populations with borderline qFIT results. Therefore, a screening strategy that combines qFIT with microbial markers could optimise the existing programme and potentially reduce the number of unnecessary diagnostic colonoscopies [15]. Though it has been reported that proteomics could also be used as biomarkers for application in stool-based CRC screening, proteins identified for detection of colorectal adenomas are mainly makers of blood in the stool and therefore have limited complementary value to hemoglobin [16]. The independence of microbial markers to faecal hemoglobin reflects its potential in improving the current qFIT-based CRC screening strategies relative to protein markers [17].

In view of rapidly evolving in understanding the role of microbiota in benign and malignant colorectal neoplasia and their use as predictors for risk prediction/stratification, we set out to provide a comprehensive and current assessment of the literature. Here, we aimed to systematically review studies investigating associations between microbial markers and colorectal neoplasia and their application for risk prediction/stratification. We additionally conducted a comparative syntheses between the identified microbial markers and the predictors employed in risk prediction models to examine to what extent predictive models include the most influential factors.

## Methods

### Study design

This study was conceived and conducted in accordance with the Preferred Reporting Items for Systematic Reviews and Meta-Analyses (PRISMA) statement [18]. The study protocol was registered in PROSPERO (registration number: CRD42021227165).

### Literature search and screening

We conducted a systematic literature search in MEDLINE and EMBASE databases (both through the OVID interface) from inception to December 1, 2020 to identify all relevant studies. No restrictions were applied for the literature searches. The detailed search syntax is presented in Supplementary Table S1. Title, abstract and full text were screened independently by two authors (L.Y. and G.Z.) based on the inclusion and exclusion criteria. Any disagreement was discussed with a senior investigator (L.W.). We also cross-checked the reference list of each eligible article for any additional studies.

### Inclusion criteria

Studies were eligible for inclusion if they met the following predefined criteria: (i) observational studies exploring the associations between microbiota and colorectal neoplasia in population-based settings; (ii) studies developing or validating prediction models for colorectal neoplasia detection or prognostication (i.e., metastasis, recurrence or survival) using microbiota-related biomarkers. The exclusion criteria were as follows: (i) studies with very small sample size (*n* < 10) were excluded due to limited statistical power and low reliability of study findings; (ii) studies published in letter or abstract forms or with no full text available were excluded as they did not include enough data for our review; (iii) studies that investigated the efficiency of probiotics or therapeutic procedures of CRC or adenoma and prediction studies in which microbiome was not included as a predictor were excluded; (iv) animal, in vitro, and in vivo experiments were all excluded. When more than one study was conducted using the same sequencing data, we chose the study with the most comprehensive information.

### Data extraction

For each included observational study, the following items were extracted: year of publication, study design, number of cases and controls, reason for colonoscopy, sample collection, antibiotic use prior to stool sample, microbiome detection method, database used for taxonomy assignment, storage temperature, microbial markers, and clinical outcome (incidence, prognosis, overall survival).

### Quality assessment

The quality of observational studies was evaluated by using the Newcastle-Ottawa Scale (NOS) [19], which is designed to assess the quality of case-control studies. For risk prediction studies, we appraised each model using the checklist for critical appraisal and data extraction of systematic reviews of prediction modelling studies (CHARMS) [20]. According to this checklist, the risk of bias for each model was assessed following the criteria, which included five domains: participant selection, measurement and reporting of predictors, definition and measurement of the outcome, attrition (loss to follow-up), data analysis. For each domain, risk of bias was classified as ‘low’ if bias was unlikely, as ‘moderate’ if the criteria for low risk were not satisfied but no fatal flaws were present, or as ‘high’ if critical flaws were identified. Owning to the extensive heterogeneities among included studies, we did not conduct any quantitative analysis. Instead, we performed descriptive syntheses and reported the results narratively and thematically.

## Results

### Literature review and study characteristics

**Fig. 1:**
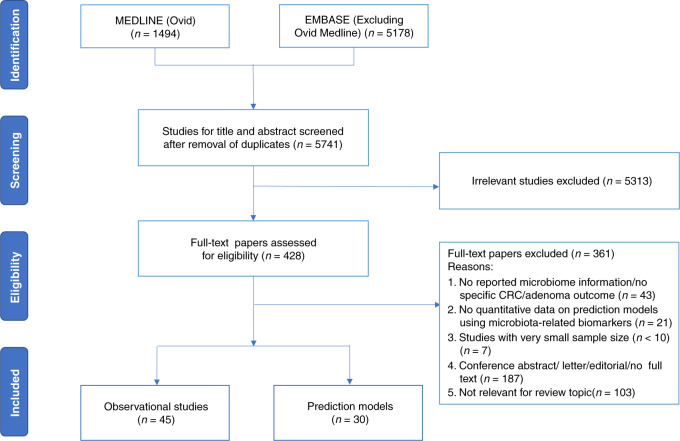
PRISMA diagram. Flowchart of the selection of studies.

Overall, the literature search retrieved 5741 unique publications across the two databases. After parallel review, a total of 45 eligible observational studies [9, 15, 21–63] exploring the associations between microbiota and colorectal neoplasia risk in population screening settings and 30 studies [15, 29, 46, 50, 53, 54, 58, 59, 64–85] developing or validating prediction models for colorectal neoplasia detection or prognostication using microbial biomarkers were included. The detailed process of study selection is documented in Fig. 1. The characteristics of included studies are presented in Table 1 and Supplementary Table S2.

**Table 1:** Summarized characteristics of 45 eligible observational studies.

Characteristics	Number of studies (%)
*Participant region*	
Europe	10 (22.2)
Asia	26 (57.8)
America	8 (17.8)
Other	1 (2.2)
*Study design*	
Prospective study	20 (44.4)
Retrospective study	25 (55.6)
*Outcome*	
Diagnosis	36 (80.0)
Metastasis	5 (11.1)
Survival	4 (8.9)
*Reason for colonoscopy*	
Routine screening	26 (57.8)
qFIT/gFOBT positive	5 (11.1)
Symptoms + Screening	5 (11.1)
Patients recruited at hospital	9 (20.0)
Antibiotic use prior to sample	
Not in 1–4 weeks	7 (15.6)
Not in 4–24 weeks	18 (40.0)
At the time of baseline assessment	3 (6.7)
Not mention	17 (37.7)
*Sample collection*	
Faecal samples	36 (70.0)
Tissue samples	6 (13.3)
Oral samples	1 (2.2)
Blood samples	2 (4.4)
*Database used for taxonomy assignment*	
RDP	15 (33.3)
Silva	6 (13.3)
Greengenes	5 (11.1)
Other	5 (11.1)
Not mention	14 (31.2)

### Microbiota markers related to colorectal neoplasia

There were 36 studies [15, 21–41, 46–54, 58–62] examining the microbiota differences for colorectal neoplasia risk, five [42, 43, 55, 56, 63] for CRC metastasis and four [9, 44, 45, 57] for CRC survival. Quality assessment using the NOS criteria classified 6.6% of included studies as low quality (NOS score: 0-5), 35.6% as moderate quality (NOS score: 6–7) and 57.8% as high quality (NOS score: 8–9). More details of the NOS assessment are presented in Supplementary Table S3.

We summarised the faecal microbial markers which were reported to be significantly different in abundance between case and control groups in at least two studies, and the results are presented in Table 2. Overall, bacteria from 18 genera belonging to five different phyla have been examined for their associations with colorectal neoplasia. At genus or species level, *Fusobacterium* (e.g., *Fusobacterium nucleatum*), *Porphyromonas* (e.g., *Porphyromonas asaccharolytica*), *Peptostreptococcus* (e.g., *Peptostreptococcus stomatis*) and *Actinomyces* were reported to be more abundant in CRC patients than healthy individuals in prospective studies [15, 22, 23, 25, 26, 29, 34, 36, 50, 52–54, 58, 59, 67, 75], and *Enterococcus* (e.g., *Enterococcus faecalis*), *Salmonella* (e.g., *Escherichia coli*) were reported in retrospective studies [27, 46, 49]. *Bifidobacterium* and *Roseburia* (e.g., *Roseburia faecis*) were consistently reported to be more abundant in healthy individuals than CRC patients [22, 23, 25, 26, 38, 39, 48, 51, 61, 62]. When comparing adenoma patients and healthy controls, *Fusobacterium* (e.g., *Fusobacterium nucleatum*) was consistently reported to be more abundant in adenoma patients with supporting evidence from at least two prospective studies [30, 58, 77, 81]. Conflicting evidence was reported for other identified microbial markers, including *Bacteroides*, *Prevotella*, *Lactobacillus*, *Ruminococcus*, *Faecalibacterium*, *Clostridium*, *Streptococcus*, *Phascolarctobacterium* and *Salmonella*.

**Table 2:** Bacteria found in significantly different abundance in CRC, adenomas and controls in at least two studies.

Bacteria taxonomic level				Reported to be more abundant in:		
Phylum	Order/Famliy	Genus	Species	CRC	Adenomas	Controls
Actinobacteria				[24]	[35]	[31]
	Bifidobacteriaceae	Bifidobacterium				[22, 38, 39, 48, 61, 62]
	[Actinomycetaceae]	Actinomyces		[34, 36]	[35]	
	[Coriobacteriaceae]	Atopobium		[34]	[36]	
			Eggerthella lenta	[25, 51]		
Bacteroidetes				[21, 24, 26, 48, 49]	[31, 37]	[24, 26, 37, 47]
	Porphyromonadaceae	Porphyromonas		[22, 26, 29]	[39]	
			Porphyromonas asaccharolytica	[15, 25, 50, 59]		
	[Bacteroidaceae]	Bacteroides		[28, 29]		[23, 26]
			Bacteroides fragilis	[34]	[30, 39]	[61]
	[Prevotellaceae]	Prevotella		[28]		[23]
Firmicutes				[26, 29, 48]	[35]	[21, 24, 26, 31, 35, 37, 47]
	Ruminococcaceae	Ruminococcus		[23, 38]	[29]	[48]
		Faecalibacterium	Faecalibacterium prausnitzii	[43]		[69]
	[Clostridiaceae]	Clostridium		[23]	[29]	
			Clostridium symbiosum	[52, 58, 69]	[58]	
	Streptococcaceae	Streptococcus		[23, 26]	[35]	[24, 48]
	[Lachnospiraceae]	Coprococcus		[50]	[53]	[53]
		Lactobacillus		[28]		[39]
		Roseburia				[23, 26, 39]
			Roseburia faecis			[25, 51]
	[Enterococcaceae]	Enterococcus		[26, 32]		
			Enterococcus faecalis	[46]	[30, 77]	
	[Peptostreptococcaceae]	Peptostreptococcus		[22, 23, 26, 36]		
			Peptostreptococcus stomatis	[15, 34, 52, 59]		
	[Acidaminococcaceae]	Phascolarctobacterium		[29]	[35]	[38]
Fusobacteria				[29, 35, 38]		
	[Fusobacteriaceae]	Fusobacterium		[22, 23, 32, 34, 36, 50, 54, 67]	[36]	
			Fusobacterium nucleatum	[15, 34, 43, 52–54, 58, 59, 75, 79]	[30, 58, 77, 81]	
Tenericutes				[26]	[35]	[35]
	Alcaligenaceae				[22, 35]	[27]
	Enterobacteriaceae	Salmonella		[27, 46]		[36]
			Escherichia coli	[43, 49]		
	[Pseudomonadaceae]	Pseudomonas		[61]	[29]	

Bacteria in square brackets were not reported on this level and are there for reference.

The number represents the corresponding order of the sited reference in the manuscript.

We identified three studies looking for microbial markers related to colorectal neoplasia beyond faecal microbiota [24, 27, 33]. There were two population-based studies assessing the association of bacterial antibody levels with CRC risk in blood samples [27, 33]. In a prospective setting, a serological study demonstrated a positive association between antibody responses to *Streptococcus gallolyticus subspecies gallolyticus* (SGG) proteins and CRC risk using pre-diagnostic blood samples [33]. Another study found serum *Salmonella antiflagellin* antibody levels to be significantly higher in CRC cases and in all cases combined (CRC + polyps) comparing to controls without polyps [27]. Using oral rinse samples, a prospective study investigated the oral microbiome and found two oral pathogens, *Treponema denticola* and *Prevotella intermedia*, to be associated with subsequent risk of CRC [24].

Associations between microbial markers and CRC prognosis (e.g., metastasis and survival) were examined in nine studies [9, 42–45, 55–57, 63]. At genus or species levels, *Fusobacterium nucleatum* and *Bacteroides fragilis* were consistently reported to be associated with CRC metastasis [43, 56]. When comparing the differentially enriched microbial markers related to CRC survival, genera *Bacteroides* and *Fusobacterium* were consistently reported to be more abundant in CRC patients with poor survival outcomes [9, 44, 45, 57].

### Multi-bacteria models for detection of colorectal neoplasia

For prediction models, 30 articles were identified describing 57 models, including seven external validation studies [50, 53, 66, 70, 71, 73, 74]. The detailed criteria and scores on risks of bias for each domain are presented in Supplementary Table S4–5 and Supplementary results. A summary of the study characteristics is presented in Table 3.

**Table 3:** Multi-bacteria models for detection of colorectal cancer and adenomas.

Author, year	Predictors	Sample examined (CRC/Adenomas/Controls)	Performance of AUROCs (CI)	Internal validation	External validation
*Diagnosis/CRC vs HC*					
Amitay, 2017 Germany	Fusobacterium nucleatum	46/223/231	0.67 (0.59–0.76)		
	Fusobacterium nucleatum + age + sex	46/223/231	0.75 (0.68–0.83)		
Baxter, 2016 Canada + USA	32 OTUs	101/162/141	0.85		
	28 OTUs + qFIT	101/162/141	0.83		
Gao, 2020 China	18 genera	100/110/332	0.86 (0.78–0.93)	Validation cohort	
	18 genera + qFIT	100/110/332	0.99 (0.98–1.00)		
Zackular, 2014 USA	6 OTUs	30/30/30	0.80 (0.69–0.91)		
	6OTUs + age + race + BMI	30/30/30	0.92 (0.86–0.99)		
Coker, 2020 China	9 species	73/NA/92	0.82 (0.70–0.94)	Chinese Cohort C2	
Alomair, 2018 Saudi Arabia	11 genera	29/NA/29	0.89		
Zhang, 2020 China	5 oral microbiome OTUs	161/NA/58	0.84 (0.77–0.90)		
Arabameri, 2018 France	22 species	53/27/61	0.91		American cohort & Austrian cohort
	22 species + gFOBT	53/27/61	0.92		
Liang, 2019 China	Fusobacterium nucleatum	170/NA/200	0.87 (0.83–0.90)		Shanghai cohort II
	Fusobacterium nucleatum + qFIT	170/NA/200	0.92 (0.82–0.96)		
	4 bacteria	170/NA/200	0.89 (0.85–0.92)		
Baxter, 2016 Canada + USA	34 OTUs	120/198/172	0.85		
	23 OTUs + qFIT	120/198/172	0.95		
Guo, 2018 China	Fusobacterium nucleatum	215/NA/156	0.88		Cohort II
	Fn/Fp+Fn/Bb	215/NA/156	0.94		
Tarallo, 2019 Italy	bsRNA + bDNA + hsa-miRNAs	29/27/24	0.87		
Flemer, 2017 Ireland	16 faecal microbiota OTUs	99/32/103	0.81 (0.73–0.81)		
	16 oral microbiota OTUs	99/32/103	0.90 (0.83–0.90)		
	29 oral OTUs + 34 fecal OTUs	99/32/103	0.94 (0.87–0.94)		
Ai, 2017 China	6 species	42/47/52	0.94		
	6 species + gFOBT	42/47/52	0.95		French cohort
Ai, 2019 China	9 genera	53/42/61	0.93		French cohort & Austria cohort
Yachida, 2019 Japan	29 species	365/NA/251	0.73*		
	55 species	365/NA/251	0.83		
Zeller, 2014 France	22 species	53/42/61	0.84*		Denmark cohort & Spain cohort & Germany cohort
	22 species + gFOBT	53/42/61	0.87*		
Kim, 2020 Korea	Collinsella + Solanum melongena	32/NA/40	0.95		
	Collinsella + Solanum melongena + leucine + oxalic acid	32/NA/40	1.00		
Guven, 2019 Belgium	Streptococcus gallolyticus	71/NA/77	0.84 (0.72–0.96)		
Yu, 2017 China	20 microbial gene markers	74/NA/54	0.71	Chinese Cohort C2	Danish cohort & French cohort & Austrain cohort
Liang, 2020 China	4 genera	13/NA/22	0.83		
Shen, 2020 China	Firmicutes cluster1 (IVF group)	30/NA/25	0.93		
	Fusobacteria cluster	30/NA/25	0.94		
Xie, 2017 China	Clostridium symbiosum + qFIT	327/212/242	0.84* (0.77–0.89)		
	Clostridium symbiosum + Fusobacteria nucleatum + qFIT + CEA	327/212/242	0.86* (0.79–0.91)		
	Clostridium symbiosum + Fusobacteria nucleatum + qFIT + CEA	327/212/242	0.90 (0.87–0.93)		
Wang, 2016 China	Fusobacterium nucleatum + CEA	258/NA/200	0.85		
*Diagnosis/Adenomas vs HC*					
Gao, 2020 China	18 genera	100/110/332	0.62 (0.52–0.71)	Validation cohort	
	18 genera + qFIT	100/110/332	0.72 (0.63–0.81)		
Zackular, 2014 USA	5 OTUs	30/30/30	0.84 (0.74–0.94)		
	5 OTUs + age + race + BMI	30/30/30	0.90 (0.82–0.98)		
Flemer, 2017 Ireland	12 oral microbiota OTUs	99/32/103	0.89 (0.80–0.89)		
	12 oral OTUs + 16 faecal OTUs	99/32/103	0.98 (0.95–0.98)		
Baxter, 2016 Canada + USA	22 OTUs	120/198/172	0.67		
	23 OTUs + qFIT	120/198/172	0.76		
Liu, 2020 China	Escherichia-Shigella + Acinetobacter	NA/22/19	0.81	Validation cohort	
	Escherichia-Shigella + Acinetobacter + BMI	NA/22/19	0.94		
Tarallo, 2019 Italy	bsRNA + bDNA + hsa-miRNAs	29/27/24	0.47		
Zhang, 2020 China	5 oral microbiome OTUs	NA/34/58	0.95 (0.91–0.99)		
Goedert, 2015 China	5 phyla + 7 genera	2/20/24	0.77		
Wei, 2020 China	2 species	36/43/53	0.79		
	Fusobacterium mortiferum + gFOBT	36/43/53	0.47		
*Prognostication*					
Jin, 2019 China	10 species	161/NA/NA	0.72 (0.59–0.88)		
Li, 2019 China	3 species	37/NA/NA	0.82 (0.69–0.96)		
	3 species + age	37/NA/NA	0.91 (0.81–1.00)		
Yu, 2019 China	3 genera	20/NA/NA	0.79 (0.63–0.90)		

*qFIT* quantitative faecal immunochemical test, *gFOBT* guaiac faecal occult blood test, *OTUs* operational taxonomic units, *AUROC* area under the receiver operating characteristic curve, *BMI* body mass index, *IVF* intestinal lavage fluid, *Fn* Fusobacterium nucleatum, *Fp* Faecalibacterium prausnitzii, *Bb* Bifidobacterium.

*Early-stage detection of Colorectal Cancer.

**Fig. 2:**
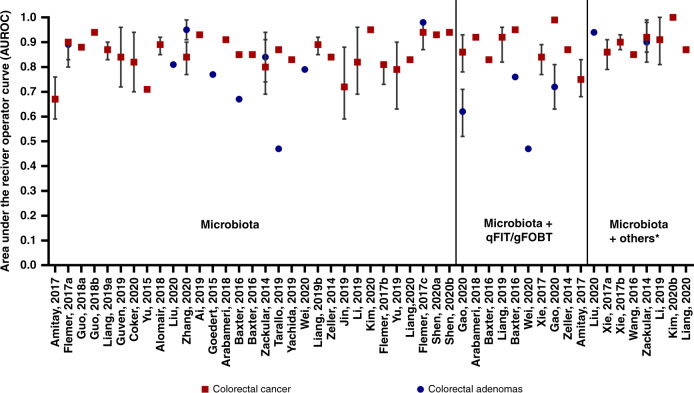
Model discrimination. Relative discriminative performance of the AUROCs (Area under the receiver operator curves) ordered by number of variables included. (*: age, BMI, race, CEA).

For CRC risk, prediction models were developed using different bacteria at different taxonomy levels, and discriminatory ability varied largely based on multiple markers. Five preditive [53, 54, 69, 74, 80] models including a single microbial marker achieved an AUROC ranging between 0.67–0.94. There were three studies [53, 54, 74] using single bacterium, *Fusobacterium nucleatum*, to distinguish CRC and healthy controls, reporting an AUROC of 0.87 (95%CI: 0.83–0.90), 0.67 and 0.88, respectively. In contrast, models using multiple bacterial species had relatively better performance as shown in Fig. 2. There were eight [15, 50, 53, 58, 59, 65, 71, 73] studies that combined faecal microbial markers plus qFIT/ guaiac faecal occult blood test (gFOBT) test result for CRC prediction. Generally, faecal microbial markers were shown to strengthen the accuracy of qFIT/gFOBT and improved the sensitivity and specificity of CRC prediction [50, 53, 59, 65, 71]. In addition, there was one study utilising *Clostridium symbiosum* and *Fusobacteria nucleatum* in combination with CEA, which achieved a good performance of 0.90 (95%CI: 0.87–0.93) for CRC discrimination [58].

Table 3 presents eight models based on multiple microbial markers to distinguish adenomas from healthy controls. Gao et al. [65] used only 18 different faecal genera and reported an AUROC of 0.86 (95%CI: 0.78–0.93) for CRC and 0.62 (95%CI: 0.52–0.71) for adenomas. Baxter et al. [15] developed two models, one containing 22 OTUs only and the other combining the OTUs with qFIT, and reported an AUROC of 0.67 and 0.76 for adenomas, respectively. Although combining microbial markers and qFIT improved the model performance, these models still had a lower sensitivity and specificity for adenoma prediction.

For prognostication of CRC, we identified three prediction studies [82–84]. One study by Li et al. [83] used three microbial markers to predict the anastomosis healing status in patients after CRC radical resection and reported an AUROC of 0.82 (95%CI: 0.69–0.96) for distinguishing the CRC patients that healed well compared to those that did not. Furthermore, there were two studies focusing on microbial prediction of adenomas recurrence in postoperative CRC patients. In one study [84], the microbiota signature of *Parabacteroides*, *Streptococcus*, and *Ruminococcus* showed an optimal discriminating performance of postoperative status with AUROC of 0.79 (95%CI: 0.63–0.90). Another study using 10 different species as predictors achieved an AUROC value of 0.72 (95%CI: 0.59–0.88) to distinguish postoperative patients with or without newly developed adenomas [82]. These results indicated that microbial makers may be clinically predictive for the prognostication of colorectal neoplasia.

Evidence was obtained by seven studies using external validation of multiple microbial models predicting colorectal neoplasia [50, 53, 66, 70, 71, 73, 74]. The model by Guo et al. which included three species markers reported an AUROC of 0.94 in a Chinese test population of 371 samples, and the same model was successfully validated in another independent Chinese cohort with an AUROC of 0.96 [74]. The prediction models developed by other five studies showed relatively lower discrimination in external validation populations. For instance, Arabameri et al. [73] used faecal metagenomes from 141 individuals attending routine CRC screening in a French cohort with an AUROC of 0.91, and the model was validated in independent American and Austrian cohorts, with lower AUROC values of 0.81 and 0.85, respectively. Liang et al. developed two models, one containing the *Fusobacterium nucleatum* and the other a combination of four bacteria, reporting AUROCs of 0.87 (95%CI: 0.83–0.90) and 0.89 (95%CI: 0.85–0.92), and received lower AUROCs of 0.68 (95%CI: 0.55–0.80) and 0.76 (95%CI: 0.64–0.87) in a smaller Chinese cohort that was used for external validation [53].

When comparing to microbial markers reported in association studies, four of the 18 genera, namely *Fusobacterium* (e.g., *Fusobacterium nucleatum*), *Peptostreptococcus* (e.g., *Peptostreptococcus stomatis*), *Porphyromonas* (e.g., *Porphyromonas asaccharolytica*) and *Clostridium* (*Clostridium symbiosum*), were commonly used predictors in prediction studies. Meanwhile, the *Fusobacterium* genus was also the most frequently used marker in CRC prognostic models.

## Discussion

In total, 45 association studies and 30 prediction studies were included in this systematic review. The included studies followed different protocols in terms of study population selection, sample collection and storage, microbiome sequencing, and databases used for taxonomy assignment. A large number of parameters were used to describe the composition of microbiome at different taxonomic levels, making it difficult to synthesise studies using meta-analysis. We, therefore, systematically reviewed the methodology and results of the included studies and summarised the microbial markers associated with colorectal neoplasia and their application for the risk prediction.

### Multiple-site microbiome for detection of colorectal neoplasia

We found seven faecal microbiota markers (e.g., *Fusobacterium*, *Enterococcus*, *Porphyromonas*, *Salmonella*, *Pseudomonas*, *Peptostreptococcus* and *Actinomyces*) at genus level that were consistently reported to be enriched in CRC patients, while two faecal microbial markers (*Bifidobacterium* and *Roseburia*) were consistently reported to be enriched in healthy controls. The reported bacterial differences between adenoma patients and healthy controls were not as consistent as with CRC. Of these, only *Fusobacterium* (e.g., *Fusobacterium nucleatum*) and *Pseudomonas* were consistently reported to be enriched in adenoma patients, indicating that these two bacteria species exhibited a progressive increase in abundance across the early to late stages of carcinogenesis. Apart from the faecal microbiome, there were also studies investigating IgG, indicating that serum antibody levels response to these specific bacteria were associated with CRC. Multiple studies indicated that CRC patients had higher levels of antibodies against *Fusobacterium nucleatum* when compared to healthy controls [85]. Furthermore, a positive association of CRC with serum antibody responses to SGG was observed in a nested case-control study, indicating CRC-related microbiota might induce specific humoral antibody and multiplex serology tests might be a new potential way for CRC detection. Oral microbiome composition was also investigated in related to CRC risk. Two oral pathogens, *Treponema denticola* and *Prevotella intermedia*, were associated with subsequent CRC risk. Findings from these studies implicated easier ways to obtain microbial markers related to CRC risk. In addition, these results raise the possibility that the oral microbiome may play an important role in CRC aetiology supporting the theory that the inflammation in gut could be driven by oral microbiota. Further studies with larger sample size are needed to confirm the identified associations and estimate the potential utilisation of the oral microbiota for CRC early detection or prevention.

### Microbiome for prognostication of colorectal neoplasia

Apart from their potential for CRC diagnosis, associations identified between gut microbiota and clinical outcomes of CRC have raised the possibility of using them as prognostic markers. A number of studies have shown that *Fusobacterium nucleatum* and *Bacteroides fragilis* are associated with CRC prognosis, and the increased abundance of these two species indicates poor survival outcome for CRC patients [9, 44]. These findings highlight the potential of quantifying *Fusobacterium nucleatum* and *Bacteroides fragilis* in tumour tissue as prognostic markers, and indicate that reducing the abundance of these bacteria might improve prognosis and survival. Nevertheless, it should be noted that their association with prognostication could be confounded by other factors like clinicopathological parameters (e.g., TNM stage), and more validation studies are needed before these biomarkers could be used in the clinical context.

### Diagnosis and prognostication of colorectal neoplasia prediction

Findings from observational studies pinpoint a potential core set of bacteria that could be used as predictive biomarkers for the detection of colorectal neoplasia. Thirty studies developed microbial prediction models for colorectal neoplasia. Faecal microbiome analysis discerned patients with CRC with varying levels of accuracy (with AUROC ranging from 0.71 to 0.95 in validation studies), but only seven of the identified models were validated in external populations. Several studies have utilised multiple bacterial species to distinguish CRC patients from healthy individuals, including three prospective studies [59, 74, 77] with large sample size (*n* > 300) achieving AUROCs of 0.85–0.94. The AUROCs reported in multiple predictor models for adenomas detection were lower than those for CRC discrimination. Combining the faecal microbiome data with other risk factors or results of screening qFIT/gFOBT tests increased the accuracy of discrimination for both CRC and adenomas. For instance, addition of faecal microbiota OTUs to qFIT or gFOBT testing improved the sensitivity for detection of CRC and advanced adenomas [15]. Findings from these predictive models indicated microbial markers have the potential to complement established tests such as gFOBT or qFIT as a non-invasive early detection tool for CRC and its precursors.

### Synthesis of results and limitations

The absence of a gold-standard unified protocol leads to great heterogeneity in study design and methodology, which limits the validity, generalizability and comparability of results reported in the included studies. The main sources of bias stemmed from methodological limitations in study population selection, sample collection and data analysis. Several recent studies indicate that there are significant variations in the gut microbiome due to differences in ethnicity, geographic location, lifestyle, nutrition, and medication use across study populations [86–89]. The “core microbiota” could be influenced by the gut environment (e.g., intestinal immune system) and colorectal neoplasia may influence the microbial community composition in reverse [90], therefore, we could not infer a causal association between identified microbiota and colorectal neoplasia based on the current evidence. It is thought that the organization of bacterial communities into biofilms (higher-order spatial structures of bacterial species) may be necessary for bacteria-induced CRC initiation [91, 92]. A previous study by Li et al. demonstrated that poly-microbial biofilms might promote pro-carcinogenic activities that may partially underlie progression along the adenoma-CRC sequence [93]. Oral antibiotics may affect the microbiome composition [94], possibly leading to chronic inflammation and tumour progression [95, 96], and the pattern of use, formulations and dosages of the drugs may have changed over time, complicating the interpretation of results. Seventeen studies did not address antibiotics taken by the participants, and three studies only excluded participants taking antibiotics at the time of recruitment which did not give enough time for the gut microbial community to return to its normal composition. Only one study was based on a population-wide CRC screening programme using fresh stool samples collected within days for microbiome analysis [61]. The majority of included studies used frozen stool sample, which were stored for a few years before analysis, where collection methods, storage temperatures and duration before analysis of faecal samples varied widely and may have a differentiating effect on the results of microbiome analysis. Sex hormones status especially oestrogen receptor beta may be another factor affecting incidence and mortality of CRC [97, 98]. However, observational studies included in this systematic review did not report the association between microbiome and colorectal neoplasia by sex, and therefore we were unable to examine any sex differences. Additionally, the included studies used three different reference databases (i.e., Silva, Ribosomal Database Project (RDP), and Greengenes database) for taxonomic assignments, which may affect the accuracy and resolution of their findings.

The identified prediction studies used different microbial features to construct their models. It is unclear to what extent the heterogeneity among studies reflects the true differences in the ability to detect CRC based on different microbial patterns or whether it reflects variations in the technical aspects of studies. It should also be noted that prediction models developed from case-control studies were not validated externally in prospective studies. Limitations identified through the quality assessment of the included studies require cautious interpretation of the reported findings.

## Conclusions

In summary, this systematic review provided a comprehensive overview of the microbial markers from multiple sites (faecal, oral and blood) for their associations with the risk of colorectal neoplasia, and summarised the evidence for applying these markers for colorectal neoplasia risk prediction and prognostication. Based on the currently published data, there is encouraging evidence that microbial markers from faecal, oral or blood specimens may be used to develop new, non-invasive and inexpensive tests that could complement the repertoire of current non-invasive CRC screening tools on their own or in combination with qFIT or gFOBT screening tests. However, current prediction models are mostly developed from case-control studies, which require further external validation in high-quality prospective studies. Future research should focus on developing unified documented and reproducible protocols for studying the human gut microbiome so that results can be more comparable and conclusions can be drawn on a larger basis. Other practical issues must be evaluated before microbiome analysis can be used in CRC screening, such as determination of cost effectiveness, affordability, and acceptability by patients and physicians, compared with established screening strategies. Collectively, these research advances have provided an unprecedented opportunity to move microbiota discoveries towards clinical applications, including prevention and treatment.

## Supplementary information

Supplementary material

## Data Availability

All data relevant to the study are included in the article or uploaded as supplementary information.
