# Supplementary material for: A systematic review of microbial markers for risk prediction of colorectal neoplasia
Source: Br J Cancer. 2022 Mar 15;126(9):1318–28. doi: 10.1038/s41416-022-01740-7 (PMC9042911; doi:10.1038/s41416-022-01740-7)
Supplement: Supplementary file 1 — Supplementary material [file 41416_2022_1740_MOESM1_ESM.docx]

**SUPPLEMENTARY MATERIAL**

**Table. S1** Search strategy.

**Table. S2** Characteristics of observational studies included in the systematic review.

**Table. S3** Quality assessment by means of Newcastle-Ottawa Scale (NOS) tool for 45 included observational studies.

**Table. S4** Criteria for scoring of risk of bias using the CHARMS checklist.

**Table. S5** Risk of bias assessment for the evidence represented in 30 prediction models.

**Table. S1** Search strategy

| MEDLINE (OVID)  1. (colorectal) OR (rectal) OR (rectum) OR (colonic) OR (colon) OR (colorectal) OR (bowel) OR (sigmoid) OR (intestin).mp. [mp=title, abstract, heading word, drug trade name, original title, device manufacturer, drug manufacturer, device trade name, keyword, floating subheading word, candidate term word]  2. (cancer) OR (carcinoma) OR (neoplas) OR (tumor) OR (adeno) OR (carcinoma) OR (adenom) OR (lesion) OR (CRC).mp. [mp=title, abstract, heading word, drug trade name, original title, device manufacturer, drug manufacturer, device trade name, keyword, floating subheading word, candidate term word]  3. Bacteria/ OR circulating bacteria.mp.  4. Mouth/ OR oral microbiota.mp. OR Microbiota/  5. Mouth/ OR oral microflora.mp.  6. Gastrointestinal Microbiome/  7. Fecal microbiome.mp. OR Gastrointestinal Microbiome/  8. 1 AND 2  9. 3 OR 4 OR 5 OR 6 OR 7  10. 8 AND 9 |
| --- |
| EMBASE (OVID)  1. (colorectal or rectal or rectum or colonic or colon or colorectal or bowel or sigmoid or intestin).mp. [mp=title, abstract, heading word, drug trade name, original title, device manufacturer, drug manufacturer, device trade name, keyword, floating subheading word, candidate term word]  2. (cancer or carcinoma or neoplas or tumor or adeno or carcinoma or adenom or lesion or CRC).mp. [mp=title, abstract, heading word, drug trade name, original title, device manufacturer, drug manufacturer, device trade name, keyword, floating subheading word, candidate term word]  3. Bacteria/ or circulating bacteria.mp.  4. Mouth/ or oral microbiota.mp. or Microbiota/  5. Mouth/ or oral microflora.mp.  6. Gastrointestinal Microbiome/  7. Fecal microbiome.mp. or Gastrointestinal Microbiome/  8. 1 and 2  9. 3 or 4 or 5 or 6 or 7  10. 8 and 9 |

**Table. S2 Characteristics of observational studies included in the systematic review.**

| **Author, Year/Country** | **Study participants Age (Mean / Median)** | | | | | | **Reason for colonoscopy** | **Sample collection** | **Antibiotic use prior to stool sample** | **Microbiome detection Method** | **Database used for  taxonomy assignment** | **Temperature for storage** | **Groups compared for bacterial differences** |
| --- | --- | --- | --- | --- | --- | --- | --- | --- | --- | --- | --- | --- | --- |
|  | **CRC** | | **Adenomas** | | **Controls** | |  |  |  |  |  |  |  |
|  | No. | Median/  Mean | No. | Median/Mean | No. | Median/Mean |  |  |  |  |  |  |  |
|  |  |  |  |  |  |  | **Diagnosis** |  |  |  |  |  |  |
| Chen, 2020 China | 25 | 50.60 | - | - | 25 | 61.64 | Screening | Faecal samples | Not in 3 months | 16S rRNA | NCBI database | -80℃ | CRC vs HC |
| Chen, 2012a China | 32 | 65 | - | - | 34 | 56 | Screening | Gut swab | Not in 1 months | 16S rRNA, regions V1-V3 | Silva | -80℃ | CRC vs HC |
| Chen, 2012b China | 21 | 64 | - | - | 22 | 64 | Screening | Faecal samples | Not in 1 months | 16S rRNA, regions V1-V3 | Silva | -80℃ | CRC vs HC |
| Wang, 2017 China | 15 | 52.5 | - | - | 12 | [no info] | Screening | Faecal samples | Not in 1 months | 16S rRNA, regions V4 | [no info] | -80℃ | CRC vs HC |
| Yang, 2019 China | 50 | 60 | - | - | 50 | 60 | Screening after positive FOBT | Faecal samples | Not in 2 months | 16S rRNA, regions V3-V4 | RDP | -80℃ | CRC vs HC |
| Zhang, 2018 China | 130 | 60.5 | 88 | 59.6 | 130 | 58.6 | Screening | Faecal samples | Not in 6 months | 16S rDNA,  V3-V4 | RDP | -80℃ | CRC vs Adenoma or CRC vs HC |
| Ziantizadeh, 2018 Iran | 25 | 58 | - | - | 24 | 52 | Familial screening | Faecal samples | Not in 1 month | 16S rRNA | [no info] | -80℃ | CRC vs HC |
| Wang, 2012 China | 46 | 60 | - | - | 56 | 49 | Physical examination | Faecal samples | Not in 3 months | 16S rRNA, V3 | RDP | -80℃ | CRC vs HC |
| Grobbee, 2019 Netherlands | 200 | 64 | - | - | - | - | Screening after positive FIT | Faecal samples | [no info] | 16S rRNA, V3-V4 | Silva | -20℃ | CRC vs HC |
| Ocvirk, 2019 USA | - | - | 16 | [no info] | 16 | [no info] | Screening | Faecal samples | Not in 3 months | 16S rRNA, V4 | RDP | -70℃ | Adenoma vs HC |
| Ohigashi, 2012 Japan | 93 | 68.9 | 22 | 66.6 | 27 | 65.6 | Screening | Faecal samples | Exclude | 16S or 23S rRNA | [no info] | 4℃ | CRC vs HC |
| Sobhani, 2011 France | 60 | 67.1 | - | - | 119 | 55.8 | Screening + symptoms | Faecal samples | Exclude | 16S rRNA, regions V3-V4 | RDP-II | -20℃ | CRC vs HC |
| Zackular, 2014 USA | 30 | 59.4 | 30 | 61.3 | 30 | 55.3 | Screening | Faecal samples | [no info] | 16S rRNA, region V4 | Silva | -80℃ | CRC vs HC or Adenoma vs HC |
| Zeller, 2014 France | 53 | 67.75 | 42 | 65 | 61 | 63 | Screening + FOBT positive | Faecal samples | [no info] | Metagenome | Silva | -80℃ | CRC vs HC + Adenoma |
| Hale, 2016 USA | - | - | 233 | 66.5 | 547 | 66.5 | Screening | Faecal samples | Not exclude | 16s rRNA | RDP | -80℃ | Adenoma vs HC |
| Yusuf, 2016 Indonesia | 16 | 50.06 | - | - | 16 | 53.06 | Screening | Faecal samples | Exclude | 16S rRNA, regions V3 | RDP | [no info] | CRC vs HC |
| Yang, 2019 China | 31 | 61.06 | 59 | 61.12 | 104 | 60.71 | Screening | Faecal samples | Not in 2 months | 16S rRNA, regions V3-V4 | Silva | −80 °C | CRC vs adenoma |
| Chénard, 2020 Canada | 235 | 62 | - | - | 265 | 61 | Screening + FOBT positive | Faecal samples | [no info] | 16S rRNA, regions V4 | Greengenes | −80 °C | CRC vs Control |
| Kasai, 2016 Japan | 9 | 54.3 | 50 | 53.5 | 49 | 48.8 | Screening | Faecal samples | Not in current | 16S rRNA, regions V3-V4 | T-RFLP | 4℃ | CRC vs HC |
| Peters, 2016 USA | - | - | 144 | 63.1 | 323 | 61.3 | Screening + FOBT positive | Faecal samples | Not in 1 month | 16S rRNA, regions V4 | Greengenes | -80℃ | Adenoma vs HC |
| Saito, 2019 Japan | 24 | 66 | 47 | 67 | 10 | 58 | Various reasons | Faecal samples | Not exclude | Metagenome | Greengenes | -80℃ | CRC vs HC or Adenoma vs HC |
| Tunsjø, 2019 Norwegian | 25 | 70 | 25 | 69 | 22 | 57 | Scheduled + symptoms | Faecal and tissue samples | [no info] | 16S rRNA, regions V3-V4 | Greengenes | -80℃ | CRC vs HC |
| Liu, 2020 China | 51 | [no info] | 54 | [no info] | 42 | [no info] | Screening | Faecal samples | Not in 2 months | 16S rRNA, regions V4 | Greengenes/RDP | -80℃ | CRC vs HC or Adenoma vs HC |
| Sarhadi, 2020a Iranian | 52 | 62.4 | - | - | 47 | 62.8 | Symptoms + Screening | Faecal samples | [no info] | 16S rRNA, regions V2-V4, V6-V9 | [no info] | -80℃ | CRC vs HC |
| Sarhadi, 2020b Finnish | 31 | 71.9 | - | - | 13 | 44.2 | Symptoms + Screening | Faecal samples | [no info] | 16S rRNA , regions V2-V4, V6-V9 | [no info] | -80℃ | CRC vs HC |
| Rezasoltani, 2020 Iran | - | - | 29 | 58.03 | 31 | 59.84 | Screening | Faecal samples | [no info] | 16S rRNA | [no info] | -80℃ | Adenoma vs HC |
| Geravand, 2019 Iran | 25 | 54 | - | - | 24 | 50 | Screening | Faecal samples | Not in 1 month | qRT-PCR | [no info] | -20℃ | CRC vs HC |
| Liang, 2017a China | 170 | 67.2 | 97 | 60.5 | 200 | 59.3 | Symptoms + Screening | Faecal samples | Not in 3 months | 16S rRNA | [no info] | -20℃ | CRC vs HC |
| Liang, 2017b China | 33 | 63.4 | - | - | 36 | 53.2 | Symptoms + Screening | Faecal samples | Not in 3 months | 16S rRNA | [no info] | -80℃ | CRC vs HC or Adenoma vs HC |
| Amitay, 2017 German | 46 | 67 | 223 | 63 | 231 | 61 | Screening | Faecal samples | [no info] | 16S rRNA | RDP | -70℃ | CRC vs HC or Adenoma vs HC |
| Feng, 2015 Austria | 46 | 67.1 | 44 | 66.8 | 57 | 67.3 | Symptoms+ screening | Faecal samples | Not in 3 months | Metagenome | IMG | -80℃ | CRC vs HC or Adenoma vs HC |
| Rezasoltani,2018 Iran | - | - | 29 | 58.03 | 31 | 59.84 | Screening | Faecal samples | Not in 3 months | qPCR | BLAST | -80℃ | Adenoma vs HC |
| Xie, 2017, China | 327 | 63.13 | 212 | 62.61 | 242 | 59.49 | Screening | Faecal samples | Not in 1 month | 16S rRNA | [no info] | -80℃ | CRC vs HC or Adenoma vs HC |
| Baxter, 2016  Canada + USA | 101 | - | 162 | - | 141 | - | Scheduled + screening | Faecal samples | [no info] | 16S rRNA, region V4 | RDP | -80℃ | CRC vs HC or Adenoma vs HC |
| Baxter, 2016  Canada + USA | 120 | 60 | 198 | 60 | 172 | 60 | Screening | Faecal samples | [no info] | 16S rRNA, region V4 | RDP | -80℃ | CRC vs Adenoma or CRC or HC |
| Goedert, 2015 China | - | - | 24 | 65 | 24 | 65 | Screening | Faecal samples | Not exclude | 16S rRNA, region V3-V4 | RDP | -80℃ | Adenoma vs HC |
| Yang, 2019 USA | 231 | [no info] | - | - | 462 | [no info] | Screening | Oral samples | Not in 1 week | 16s rRNA, region V4 | HOMD | [no info] | CRC vs HC |
| Kato, 2015  Netherlands + USA | 70 | [no info] | - | - | 74 | [no info] | Screening | Blood sample | [no info] | ELISA | [no info] | -80℃ | CRC vs HC |
| Butt, 2018 Europe | 485 | 59 | - | - | 485 | 60 | [no info] | Blood samples | [no info] | antibody response | [no info] | -196℃ | CRC vs HC |
|  |  |  |  |  |  |  | **Metastasis** |  |  |  |  |  |  |
| Wu, 2019 China | 12 | [no info] | - | - | 18 | [no info] | Recruited at hospital | Faecal samples | Not in 3 months | 16S rRNA, V4 | Greengenes | -80℃ | LNM vs non-LNM |
| Hale, 2018 USA | 83 | [no info] | - | - | - | - | Recruited at hospital | Tissue samples | [no info] | 16S rRNA, V3-V5 | RDP | -80℃ | dMMR vs pMMR |
| Han, 2020 China | 53 | 65.75 | - | - | - | - | Recruited at hospital | Faecal samples | [no info] | 16S rRNA, V3-V4 | RDP | -80℃ | N0 vs N1 vs N2 vs NX |
| Toychiev, 2018 Tashkent | 56 | [no info] | - | - | 144 | [no info] | Recruited at hospital | Faecal samples | Not in 2-3weeks | [no info] | [no info] | [no info] | Metastasis vs no metastasis |
| Zhou, 2016 China | 97 | 64.6 | - | - | - | - | Cases: patients | Tissue samples | Not in 1 month | 16S rRNA | [no info] | -80℃ | Metastasis vs no metastasis |
|  |  |  |  |  |  |  | **Survival** |  |  |  |  |  |  |
| Ge, 2020 China | 55 | 64.47 | - | - | - | - | Recruited at hospital after follow up | Tissue samples | [no info] | 16S rRNA, V3-V4 | Silva | -80℃ | Stage III colon cancer vs Stage I/II |
| Lee, 2020 Korea | 126 | [no info] | - | - | - | - | Cases: CRC patients | Tissue samples | [no info] | 16S rRNA | [no info] | [no info] | Fn-high vs Fn low/negative |
| Mima, 2015 USA | 1069 | 69.3 | - | - | - | - | Recruited at hospital after follow up | Tissue samples | [no info] | qPCR | [no info] | [no info] | Fn-high/low vs Fn- negative |
| Wei, 2016 China | 180 | 62.2 | - | - | - | - | Recruited at hospital after follow up | Tissue samples | Not in 2 months | 16S rRNA, V4 | Greengenes + RDP | -80℃ | Worse prognosis vs prognosis group |

| **Table. S3 Quality assessment by means of Newcastle-Ottawa Scale (NOS) tool for 45 included observational studies.** | | | | | | | | | | | | | |  |
| --- | --- | --- | --- | --- | --- | --- | --- | --- | --- | --- | --- | --- | --- | --- |
| **CASE-CONTROL STUDIES** |  | Selection | | | |  | Comparability |  | Exposure | | |  | Total score (out of 9) | |
| Author, year (ref) |  | Is the case definition adequate | Representativeness of the cases | Selection of community controls | Definition of controls |  | Comparability of cases and controls on bases of the design or analysis |  | Same method of ascertainment for cases and controls | All samples collected before bowel preparation | Adjustment for multiple testing/Correction |  |  |  |
| Chen, 2020^1^ |  | 1 | 1 | 1 | 1 |  | 2 |  | 1 | 1 | 0 |  | 8 | |
| Chen, 2012^2^ |  | 1 | 1 | 0 | 1 |  | 2 |  | 1 | 1 | 0 |  | 7 | |
| Wang, 2017^3^ |  | 1 | 1 | 0 | 1 |  | 2 |  | 1 | 1 | 0 |  | 7 | |
| Yang, 2019^4^ |  | 1 | 1 | 1 | 1 |  | 2 |  | 1 | 0 | 1 |  | 8 | |
| Zhang, 2018^5^ |  | 1 | 1 | 0 | 1 |  | 2 |  | 1 | 1 | 1 |  | 8 | |
| Wang, 2012^6^ |  | 1 | 1 | 0 | 1 |  | 1 |  | 1 | 1 | 0 |  | 6 | |
| Kato, 2015^7^ |  | 1 | 1 | 0 | 1 |  | 2 |  | 1 | 0 | 1 |  | 6 | |
| Sobhani, 2011^8^ |  | 1 | 1 | 0 | 1 |  | 2 |  | 1 | 1 | 0 |  | 7 | |
| Zackular, 2014^9^ |  | 1 | 1 | 1 | 1 |  | 2 |  | 1 | 0 | 0 |  | 7 | |
| Rezasoltani,2018^10^ |  | 1 | 1 | 0 | 0 |  | 2 |  | 1 | 0 | 1 |  | 7 | |
| Hale, 2016^11^ |  | 1 | 1 | 1 | 1 |  | 2 |  | 1 | 1 | 1 |  | 9 | |
| Yang, 2019^12^ |  | 1 | 1 | 0 | 1 |  | 2 |  | 1 | 1 | 1 |  | 8 | |
| Butt, 2018^13^ |  | 1 | 1 | 1 | 1 |  | 2 |  | 1 | 0 | 1 |  | 8 | |
| Kasai, 2016^14^ |  | 1 | 1 | 1 | 1 |  | 2 |  | 1 | 1 | 0 |  | 8 | |
| Peters, 2016^15^ |  | 1 | 1 | 1 | 1 |  | 2 |  | 1 | 1 | 1 |  | 9 | |
| Saito, 2019^16^ |  | 1 | 1 | 0 | 1 |  | 2 |  | 1 | 0 | 0 |  | 6 | |
| Liu, 2020^17^ |  | 1 | 1 | 1 | 1 |  | 2 |  | 1 | 0 | 1 |  | 8 | |
| Sarhadi, 2020^18^ |  | 1 | 1 | 1 | 1 |  | 2 |  | 1 | 0 | 1 |  | 8 | |
| Rezasoltani, 2020^19^ |  | 1 | 1 | 0 | 1 |  | 2 |  | 1 | 1 | 1 |  | 8 | |
| Geravand, 2019^20^ |  | 1 | 1 | 0 | 1 |  | 2 |  | 1 | 0 | 0 |  | 6 | |
| Ziantizadeh, 2018^21^ |  | 1 | 1 | 0 | 1 |  | 2 |  | 1 | 0 | 0 |  | 6 | |
| Wu, 2019^22^ |  | 1 | 1 | 0 | 0 |  | 2 |  | 1 | 1 | 1 |  | 8 | |
| Hale, 2018^23^ |  | 1 | 0 | 0 | 0 |  | 2 |  | 1 | 0 | 1 |  | 5 | |
| Han, 2020^24^ |  | 1 | 1 | 0 | 0 |  | 2 |  | 1 | 0 | 0 |  | 5 | |
| Lee, 2020^25^ |  | 1 | 0 | 0 | 0 |  | 2 |  | 1 | 0 | 1 |  | 5 | |
| Wei, 2016^26^ |  | 1 | 1 | 0 | 0 |  | 2 |  | 1 | 0 | 1 |  | 7 | |
| Goedert, 2015^27^ |  | 1 | 0 | 1 | 1 |  | 1 |  | 1 | 1 | 1 |  | 8 | |
| Yang, 2019^28^ |  | 1 | 1 | 0 | 1 |  | 2 |  | 1 | 1 | 1 |  | 8 | |
| **COHORT STUDIES** |  | Selection | | | |  | Comparability |  | Exposure | | |  | Total score (out of 9) | |
| Author, year (ref) |  | Representativeness of the cases | Selection of the control | Ascertainment of exposure | Primary diagnosed at start of study |  | Comparability of cohort on bases of the design or analysis |  | Same method of ascertainment for cases and controls | Follow up enough for cases and controls | Adjustment for multiple testing/Correction |  |  |  |
| Feng, 2015^29^ |  | 1 | 1 | 1 | 1 |  | 2 |  | 1 | 0 | 1 |  | 8 | |
| Grobbee, 2019^30^ |  | 1 | 1 | 1 | 1 |  | 2 |  | 1 | 0 | 1 |  | 6 | |
| Zeller, 2014^31^ |  | 1 | 1 | 1 | 1 |  | 2 |  | 1 | 1 | 1 |  | 9 | |
| Chénard, 2020^32^ |  | 1 | 1 | 0 | 1 |  | 2 |  | 1 | 1 | 0 |  | 7 | |
| Tunsjø, 2019^33^ |  | 1 | 1 | 1 | 1 |  | 2 |  | 1 | 0 | 1 |  | 8 | |
| Liang, 2017^34^ |  | 1 | 1 | 1 | 1 |  | 2 |  | 1 | 1 | 1 |  | 9 | |
| Amitay, 2017^35^ |  | 1 | 1 | 1 | 1 |  | 2 |  | 1 | 1 | 1 |  | 9 | |
| Toychiev, 2018^36^ |  | 1 | 1 | 1 | 1 |  | 2 |  | 1 | 0 | 1 |  | 8 | |
| Zhou, 2016^37^ |  | 1 | 1 | 1 | 1 |  | 2 |  | 1 | 0 | 0 |  | 8 | |
| Ge, 2020^38^ |  | 1 | 0 | 1 | 1 |  | 2 |  | 1 | 1 | 1 |  | 8 | |
| Mima, 2015^39^ |  | 1 | 1 | 1 | 1 |  | 2 |  | 1 | 1 | 1 |  | 9 | |
| Xie, 2017^40^ |  | 1 | 1 | 1 | 1 |  | 2 |  | 1 | 0 | 1 |  | 8 | |
| Baxter, 2016^41^ |  | 1 | 1 | 1 | 1 |  | 2 |  | 1 | 0 | 0 |  | 7 | |
| Baxter, 2016^42^ |  | 1 | 0 | 1 | 1 |  | 2 |  | 1 | 0 | 1 |  | 7 | |
| **CROSS SECTIONAL** |  | Selection | | | |  | Comparability |  | Exposure | | |  | Total score (out of 9) | |
| Author, year (ref) |  | Representativeness of the sample | Sample size | Ascertainment of exposure | Respondents and non-respondents characteristics |  | Comparability of cohort on bases of the design or analysis |  | Same method of ascertainment for cases and controls | Statistical test | - |  |  |  |
| Ocvirk, 2019^43^ |  | 1 | 1 | 1 | 0 |  | 2 |  | 2 | 1 | - |  | 8 | |
| Ohigashi, 2012^44^ |  | 1 | 1 | 1 | 1 |  | 2 |  | 2 | 1 | - |  | 9 | |
| Yusuf, 2016^45^ |  | 1 | 0 | 1 | 0 |  | 2 |  | 1 | 1 | - |  | 6 | |

| **Table. S4 Criteria for scoring of risk of bias using the CHARMS checklist.** | | | | | |
| --- | --- | --- | --- | --- | --- |
| **Domains** | **Participants** | **Predictor** | **Outcome** | **Attrition** | **Analysis** |
| **Score for risk of bias** |  |  |  |  |  |
| **Low** | 1) Selection bias was unlikely;  2) Study avoided inappropriate inclusions or exclusions;  3) Inclusion and exclusion criteria were adequately described and participants were enrolled at a similar presentation of their disease;  4) Differences were accounted for by including appropriate predictors in the analysis. | 1) Predictor definitions were the same for all participants;  2) Predictor measurement was blinded to outcome data; 3) All predictors were available at the time the model was intended to be used; 4) Predictors were measured with valid and reproducible methods such that misclassification was limited;  5) Predictors were assessed in a similar way for all study participants. | 1) Outcome was pre-specified, measured with sufficient validity and reproducibility and measured in a similar way for all study participants; 2) Outcome was assessed independently from assessment of predictors. | 1)There was no loss-to-follow-up;  2) There were no important differences on key characteristics between included participants and those who were lost-to-follow-up or missing | 1) Relevant aspects of analysis were described allowing to judge the quality of the analysis to be adequate; 2) Missing data handled appropriately or there were no differences; 3) Predictors were included independent of p-value level; 4) Overfitting and optimism were accounted for; 5) Weights assigned according to regression coefficient  6) Calibration and discrimination were assessed; 7) Recalibration was performed or it was described that it was not needed. |
| **Moderate** | Not satisfying one of the above/ no adequate description of recruitment of study sample/ no adequate description of the sample for key predictors. | If one of the criteria was not satisfied. | If one of the criteria was not satisfied. | 1) Loss-to-follow-up was lower than 20%; 2) There were no important differences on key characteristics between included participants and those who were lost-to-follow-up or missing. | Relevant aspects of analysis were described allowing to judge the quality of the analysis to be adequate and part or none of the model evaluation items were reported. |
| **High** | If none items were adequately described. | Predictor assessment was not adequately described. | Method for assessment of outcome was not adequately described. | Loss-to-follow-up was higher than 20% and there were important differences on key characteristics between included participants;  Those who were lost-to-follow-up or missing-loss-to-follow-up was not described | Not satisfying any of the aspects under low risk of bias |

| **Table. S5 Risk of bias assessment for the evidence represented in 30 prediction models.** | | | | | | |
| --- | --- | --- | --- | --- | --- | --- |
| **Author, Year (ref)** |  | **Domain** | | | | |
|  |  | **Participants** | **Predictor** | **Outcome** | **Attrition** | **Analysis** |
| Amitay, 2017^35^ |  | L | L | L | H | M |
| Coker, 2020^46^ |  | L | L | L | H | M |
| Gao, 2020^47^ |  | L | L | L | L | L |
| Liang, 2017^34^ |  | L | L | L | H | M |
| Xie, 2017^40^ |  | L | M | M | H | M |
| Yu, 2017^48^ |  | M | L | L | H | M |
| Zackular, 2014^9^ |  | L | L | L | M | M |
| Zhang, 2020^49^ |  | L | L | L | M | H |
| Flemer, 2017^50^ |  | H | L | L | H | M |
| Guven, 2019^51^ |  | L | L | L | M | M |
| Ai, 2019^52^ |  | H | M | L | H | H |
| Ai, 2017^53^ |  | L | L | L | H | L |
| Alomair, 2018^54^ |  | L | L | L | M | M |
| Arabameri, 2018^55^ |  | H | H | H | H | M |
| Baxter, 2016^41^ |  | L | L | L | H | H |
| Baxter, 2016^42^ |  | L | L | L | H | M |
| Guo, 2018^56^ |  | L | L | L | M | H |
| Liu, 2020^57^ |  | L | L | L | L | L |
| Tarallo, 2019^58^ |  | M | L | L | H | L |
| Yachida, 2019^59^ |  | L | L | L | H | L |
| Zeller, 2014^31^ |  | L | L | L | L | L |
| Goedert, 2015^27^ |  | H | L | L | M | L |
| Kim, 2020^60^ |  | L | L | L | H | L |
| Liang,2020^61^ |  | L | L | L | H | L |
| Shen, 2020^62^ |  | L | L | L | H | H |
| Wei, 2020^63^ |  | M | L | L | M | H |
| Jin, 2019^64^ |  | L | L | L | M | H |
| Li, 2019^65^ |  | L | L | L | M | H |
| Yu, 2019^66^ |  | L | L | L | H | M |
| Wang, 2016^67^ |  | L | L | L | M | H |

**Supplementary results**

**Literature review and study characteristics**

Briefly, the sample size of included studies ranged from 27 (15 CRC cases)^3^ to 970 (485 CRC cases)^13^ . Sample collection method, storage temperature and sample type used for microbiota detection varied between studies. Thirty-six studies^1-3,5,6,8-12,14-22,24,27-36,40-45^ used frozen stool samples, two^7,13^ used frozen blood samples and one study^4^ used oral samples for microbiome detection. Twenty-five studies^1-6,8,10,12,14,15,17,20-22,26,28,29,34,36,37,40,43-45^ excluded participants that used antibiotics for a period ranging between 1 to 24 weeks prior to the time of sample collection, three studies^11,16,27^ excluded participants that were taking antibiotics at the time of recruitment, and the other seventeen studies^7,9,13,18,19,23-25,30-33,35,38,39,41,42^ did not consider antibiotics use at all. For microbiome analysis, forty studies^1-6,8-12,14,15,17-28,30,32-35,37-45^ used the 16S RNA sequencing and three studies^16,29,31^ utilised metagenome sequencing. The included studies used various databases for the assignment of taxonomy to analyze possible differences between cases diagnosed with CRC or adenomas and healthy individuals, with most using the Ribosomal Database Project (RDP)^68^ (n=15), Silva^69^ (n=6) and Greengenes^70^ (n=5) databases.

**Multi-bacteria models for detection of colorectal neoplasia**

Of them, 42 microbial prediction models were reported for CRC detection, 15 for adenomas and four for CRC prognostication. For the risk of bias assessment, the majority of the models were classified as ‘low’ risk for participant selection (76.7%), predictors (90.0%), outcome (93.3%). However, for dataset attrition, 17 studies (56.7%) were classified as ‘high’ risk, and 12 studies (40.0%) were classified as ‘moderate’ risk of bias with regards to data analysis. Predictors used these predictive models could be broadly classified as single or multiple bacterial species only (n=39), microbial markers plus qFIT/gFOBT test result (n=11), microbial markers plus basic characteristics (e.g., age, BMI, race) (n=6), and microbial markers plus carcinoma embryonic antigen (CEA) (n=2).

**Reference**

1 Chen J, Hu S, Ji D, Gao Z, Wang H, Yang Y, et al. Hemolysin BL from novel Bacillus toyonensis BV-17 induces antitumor activity both in vitro and in vivo. Gut Microbes. 2020; <http://dx.doi.org/10.1080/19490976.2020.1782158:1-15>.

2 Chen W, Liu F, Ling Z, Tong X, Xiang C. Human intestinal lumen and mucosa-associated microbiota in patients with colorectal cancer. PLoS ONE. 2012;7(6).

3 Wang X, Wang J, Rao B, Deng LI. Gut flora profiling and fecal metabolite composition of colorectal cancer patients and healthy individuals. Experimental and Therapeutic Medicine. 2017;13(6):2848-2854.

4 Yang Y, Cai Q, Shu XO, Steinwandel MD, Blot WJ, Zheng W, et al. Prospective study of oral microbiome and colorectal cancer risk in low-income and African American populations. International Journal of Cancer. 2019;144(10):2381-2389.

5 Zhang Y, Yu X, Yu E, Wang N, Cai Q, Shuai Q, et al. Changes in gut microbiota and plasma inflammatory factors across the stages of colorectal tumorigenesis: A case-control study. BMC Microbiology. 2018;18(1).

6 Wang T, Cai G, Qiu Y, Fei N, Zhang M, Pang X, et al. Structural segregation of gut microbiota between colorectal cancer patients and healthy volunteers. The Isme Journal. 2012;6(2):320-329.

7 Kato I, Boleij A, Kortman GAM, Roelofs R, Djuric Z, Severson RK, et al. Partial associations of dietary iron, smoking and intestinal bacteria with colorectal cancer risk. Nutrition and Cancer. 2013;65(2):169-177.

8 Sobhani I, Tap J, Roudot-Thoraval F, Roperch JP, Letulle S, Langella P, et al. Microbial dysbiosis in colorectal cancer (CRC) patients. PLoS ONE [Electronic Resource]. 2011;6(1):e16393.

9 Zackular JP, Rogers MA, Ruffin MTt, Schloss PD. The human gut microbiome as a screening tool for colorectal cancer. Cancer Prevention Research. 2014;7(11):1112-1121.

10 Rezasoltani S, Asadzadeh Aghdaei H, Dabiri H, Akhavan Sepahi A, Modarressi MH, Nazemalhosseini Mojarad E. The association between fecal microbiota and different types of colorectal polyp as precursors of colorectal cancer. Microbial Pathogenesis. 2018;124:244-249.

11 Hale VL, Chen J, Johnson S, Harrington SC, Yab TC, Smyrk TC, et al. Shifts in the Fecal Microbiota Associated with Adenomatous Polyps. Cancer Epidemiol Biomarkers Prev. 2017;26(1):85-94.

12 Yang TW, Lee WH, Tu SJ, Huang WC, Chen HM, Sun TH, et al. Enterotype-based Analysis of Gut Microbiota along the Conventional Adenoma-Carcinoma Colorectal Cancer Pathway. Scientific reports. 2019;9(1):10923.

13 Butt J, Jenab M, Willhauck-Fleckenstein M, Michel A, Pawlita M, Kyro C, et al. Prospective evaluation of antibody response to Streptococcus gallolyticus and risk of colorectal cancer. International Journal of Cancer. 2018;143(2):245-252.

14 Kasai C, Sugimoto K, Moritani I, Tanaka J, Oya Y, Inoue H, et al. Comparison of human gut microbiota in control subjects and patients with colorectal carcinoma in adenoma: Terminal restriction fragment length polymorphism and next-generation sequencing analyses. Oncology Reports. 2016;35(1):325-333.

15 Peters BA, Dominianni C, Shapiro JA, Church TR, Wu J, Miller G, et al. The gut microbiota in conventional and serrated precursors of colorectal cancer. Microbiome. 2016;4(1):69.

16 Saito K, Koido S, Odamaki T, Kajihara M, Kato K, Horiuchi S, et al. Metagenomic analyses of the gut microbiota associated with colorectal adenoma. PLoS ONE. 2019;14(2).

17 Liu W, Zhang R, Shu R, Yu J, Li H, Long H, et al. Study of the Relationship between Microbiome and Colorectal Cancer Susceptibility Using 16SrRNA Sequencing. BioMed Research International. 2020;2020 (no pagination).

18 Sarhadi V, Lahti L, Saberi F, Youssef O, Kokkola A, Karla T, et al. Gut microbiota and host gene mutations in colorectal cancer patients and controls of Iranian and finnish origin. Anticancer Research. 2020;40(3):1325-1334.

19 Rezasoltani S, Ghanbari R, Looha MA, Mojarad EN, Yadegar A, Stewart D, et al. Expression of main toll-like receptors in patients with different types of colorectal polyps and their relationship with gut microbiota. International Journal of Molecular Sciences. 2020;21(23):1-10.

20 Geravand M, Fallah P, Yaghoobi MH, Soleimanifar F, Farid M, Zinatizadeh N, et al. Investigation of enterococcus faecalis population in patients with polyp and colorectal cancer in comparison of healthy individuals. Arquivos de Gastroenterologia. 2019;56(2):141-145.

21 Zinatizadeh N, Khalili F, Fallah P, Farid M, Geravand M, Yaslianifard S. Potential preventive effect of Lactobacillus acidophilus and Lactobacillus plantarum in patients with polyps or colorectal cancer. Arquivos de Gastroenterologia. 2018;55(4):407-411.

22 Wu Y, Shi L, Li Q, Wu J, Peng W, Li H, et al. Microbiota Diversity in Human Colorectal Cancer Tissues Is Associated with Clinicopathological Features. Nutrition and Cancer. 2019;71(2):214-222.

23 Hale VL, Jeraldo P, Chen J, Mundy M, Yao J, Priya S, et al. Distinct microbes, metabolites, and ecologies define the microbiome in deficient and proficient mismatch repair colorectal cancers. Genome Medicine. 2018;10(1):78.

24 Han S, Wu W, Da M, Xu J, Zhuang J, Zhang L, et al. Adequate lymph node assessments and investigation of gut microorganisms and microbial metabolites in colorectal cancer. OncoTargets and Therapy. 2020;13:1893-1906.

25 Lee JA, Yoo SY, Oh HJ, Jeong S, Cho NY, Kang GH, et al. Differential immune microenvironmental features of microsatellite-unstable colorectal cancers according to Fusobacterium nucleatum status. Cancer Immunology, Immunotherapy. 2020; <http://dx.doi.org/10.1007/s00262-020-02657-x>.

26 Wei Z, Cao S, Liu S, Yao Z, Sun T, Li Y, et al. Could gut microbiota serve as prognostic biomarker associated with colorectal cancer patients' survival? A pilot study on relevant mechanism. Oncotarget. 2016;7(29):46158-46172.

27 Goedert JJ, Gong Y, Hua X, Zhong H, He Y, Peng P, et al. Fecal Microbiota Characteristics of Patients with Colorectal Adenoma Detected by Screening: A Population-based Study. EBioMedicine. 2015;2(6):597-603.

28 Yang Y, Misra BB, Liang L, Bi D, Weng W, Wu W, et al. Integrated microbiome and metabolome analysis reveals a novel interplay between commensal bacteria and metabolites in colorectal cancer. Theranostics. 2019;9(14):4101-4114.

29 Feng Q, Liang S, Jia H, Stadlmayr A, Tang L, Lan Z, et al. Gut microbiome development along the colorectal adenoma-carcinoma sequence. Nature Communications. 2015;6 (no pagination).

30 Grobbee EJ, Lam SY, Fuhler GM, Blakaj B, Konstantinov SR, Bruno MJ, et al. First steps towards combining faecal immunochemical testing with the gut microbiome in colorectal cancer screening. United European Gastroenterology Journal. 2020/no healthy control data;8(3):293-302.

31 Zeller G, Tap J, Voigt AY, Sunagawa S, Kultima JR, Costea PI, et al. Potential of fecal microbiota for early-stage detection of colorectal cancer. Molecular Systems Biology. 2014;10(11).

32 Chenard T, Malick M, Dube J, Masse E. The influence of blood on the human gut microbiome. BMC Microbiology. 2020;20(1).

33 Tunsjo HS, Gundersen G, Rangnes F, Noone JC, Endres A, Bemanian V. Detection of Fusobacterium nucleatum in stool and colonic tissues from Norwegian colorectal cancer patients. Eur J Clin Microbiol Infect Dis. 2019;38(7):1367-1376.

34 Liang Q, Chiu J, Chen Y, Huang Y, Higashimori A, Fang J, et al. Fecal Bacteria Act as Novel Biomarkers for Noninvasive Diagnosis of Colorectal Cancer. Clinical Cancer Research. 2017;23(8):2061-2070.

35 Amitay EL, Werner S, Vital M, Pieper DH, Hofler D, Gierse IJ, et al. Fusobacterium and colorectal cancer: Causal factor or passenger? Results from a large colorectal cancer screening study. Carcinogenesis. 2017;38(8):781-788.

36 Toychiev A, Abdujapparov S, Imamov A, Navruzov B, Davis N, Badalova N, et al. Intestinal helminths and protozoan infections in patients with colorectal cancer: prevalence and possible association with cancer pathogenesis. Parasitology Research. 2018;117(12):3715-3723.

37 Zhou Y, He H, Xu H, Li Y, Li Z, Du Y, et al. Association of oncogenic bacteria with colorectal cancer in South China. Oncotarget. 2016;7(49):80794-80802.

38 Ge W, Hu H, Cai W, Xu J, Hu W, Weng X, et al. High-risk Stage III colon cancer patients identified by a novel five-gene mutational signature are characterized by upregulation of IL-23A and gut bacterial translocation of the tumor microenvironment. International Journal of Cancer. 2020;146(7):2027-2035.

39 Mima K, Nishihara R, Qian ZR, Cao Y, Sukawa Y, Nowak JA, et al. Fusobacterium nucleatum in colorectal carcinoma tissue and patient prognosis. Gut. 2016;65(12):1973-1980.

40 Xie YH, Gao QY, Cai GX, Sun, Sun XM, Zou TH, et al. Fecal Clostridium symbiosum for Noninvasive Detection of Early and Advanced Colorectal Cancer: Test and Validation Studies. 2017;1:32-40.

41 Baxter NT, Koumpouras CC, Rogers MA, Ruffin MT, Schloss PD. DNA from fecal immunochemical test can replace stool for detection of colonic lesions using a microbiota-based model. Microbiome. 2016;4(1):59.

42 Baxter NT, Ruffin MT, Rogers MA, Schloss PD. Microbiota-based model improves the sensitivity of fecal immunochemical test for detecting colonic lesions. Genome medicine. 2016;8(1):37.

43 Ocvirk S, Wilson AS, Posma JM, Li JV, Koller KR, Day GM, et al. A prospective cohort analysis of gut microbial co-metabolism in Alaska Native and rural African people at high and low risk of colorectal cancer. Am J Clin Nutr. 2020;111(2):406-419.

44 Ohigashi S, Sudo K, Kobayashi D, Takahashi O, Takahashi T, Asahara T, et al. Changes of the intestinal microbiota, short chain fatty acids, and fecal pH in patients with colorectal cancer. Digestive Diseases and Sciences. 2013;58(6):1717-1726.

45 Yusuf F, Ilyas S, Damanik HA, Fatchiyah F. Microbiota Composition, HSP70 and Caspase-3 Expression as Marker for Colorectal Cancer Patients in Aceh, Indonesia. Acta medica Indonesiana. 2016;48(4):289-299.

46 Coker OO, Wu WKK, Wong SH, Sung JJY, Yu J. Altered Gut Archaea Composition and Interaction With Bacteria Are Associated With Colorectal Cancer. Gastroenterology. 2020;159(4):1459-1470.e1455.

47 Gao R, Wang Z, Li H, Cao Z, Gao Z, Chen H, et al. Gut microbiota dysbiosis signature is associated with the colorectal carcinogenesis sequence and improves the diagnosis of colorectal lesions. Journal of Gastroenterology and Hepatology. 2020; <http://dx.doi.org/10.1111/jgh.15077>.

48 Yu J, Feng Q, Wong SH, Zhang D, Yi Liang Q, Qin Y, et al. Metagenomic analysis of faecal microbiome as a tool towards targeted non-invasive biomarkers for colorectal cancer. Gut. 2015;66(1):70-78.

49 Zhang S, Kong C, Yang Y, Cai S, Li X, Cai G, et al. Human oral microbiome dysbiosis as a novel non-invasive biomarker in detection of colorectal cancer. Theranostics. 2020;10(25):11595-11606.

50 Flemer B, Warren RD, Barrett MP, Cisek K, Das A, Jeffery IB, et al. The oral microbiota in colorectal cancer is distinctive and predictive. Gut. 2018;67(8):1454-1463.

51 Guven DC, Dizdar O, Alp A, Akdogan Kittana FN, Karakoc D, Hamaloglu E, et al. Analysis of Fusobacterium nucleatum and Streptococcus gallolyticus in saliva of colorectal cancer patients. Biomark. 2019;13(9):725-735.

52 Ai D, Pan H, Li X, Gao Y, Liu G, Xia LC. Identifying gut microbiota associated with colorectal cancer using a zero-inflated lognormal model. Frontiers in Microbiology. 2019;10(APR).

53 Ai L, Tian H, Chen Z, Chen H, Xu J, Fang JY. Systematic evaluation of supervised classifiers for fecal microbiota-based prediction of colorectal cancer. Oncotarget. 2017;8(6):9546-9556.

54 Alomair AO, Masoodi I, Alyamani EJ, Allehibi AA, Qutub AN, Alsayari KN, et al. Colonic mucosal microbiota in colorectal cancer: A single-center metagenomic study in Saudi Arabia. Gastroenterology Research and Practice. 2018;2018 (no pagination).

55 Arabameri A, Asemani D, Teymourpour P. Detection of Colorectal Carcinoma Based on Microbiota Analysis Using Generalized Regression Neural Networks and Nonlinear Feature Selection. IEEE/ACM transactions on computational biology and bioinformatics. 2020;17(2):547-557.

56 Guo S, Li L, Xu B, Li M, Zeng Q, Xiao H, et al. A Simple and Novel Fecal Biomarker for Colorectal Cancer: Ratio of Fusobacterium Nucleatum to Probiotics Populations, Based on Their Antagonistic Effect. Clinical Chemistry. 2018;64(9):1327-1337.

57 Liu Y, Geng R, Liu L, Jin X, Yan W, Zhao F, et al. Gut Microbiota-Based Algorithms in the Prediction of Metachronous Adenoma in Colorectal Cancer Patients Following Surgery. Frontiers in Microbiology. 2020;11 (no pagination).

58 Tarallo S, Ferrero G, Gallo G, Francavilla A, Clerico G, Luc AR, et al. Altered fecal small RNA profiles in colorectal cancer reflect gut microbiome composition in stool samples. mSystems. 2019;4(5).

59 Yachida S, Mizutani S, Shiroma H, Shiba S, Nakajima T, Sakamoto T, et al. Metagenomic and metabolomic analyses reveal distinct stage-specific phenotypes of the gut microbiota in colorectal cancer. Nature Medicine. 2019;25(6):968-976.

60 Kim DJ, Yang J, Seo H, Lee WH, Ho Lee D, Kym S, et al. Colorectal cancer diagnostic model utilizing metagenomic and metabolomic data of stool microbial extracellular vesicles. Scientific reports. 2020;10(1):2860.

61 Liang S, Mao Y, Liao M, Xu Y, Chen Y, Huang X, et al. Gut microbiome associated with APC gene mutation in patients with intestinal adenomatous polyps. International journal of biological sciences. 2020;16(1):135-146.

62 Shen W, Sun J, Yao F, Lin K, Yuan Y, Chen Y, et al. Microbiome in Intestinal Lavage Fluid May Be A Better Indicator in Evaluating The Risk of Developing Colorectal Cancer Compared with Fecal Samples. Translational Oncology. 2020;13(5).

63 Wei PL, Hung CS, Kao YW, Lin YC, Lee CY, Chang TH, et al. Classification of changes in the fecal microbiota associated with colonic adenomatous polyps using a long-read sequencing platform. Genes. 2020;11(11):1-14.

64 Jin Y, Liu Y, Zhao L, Zhao F, Feng J, Li S, et al. Gut microbiota in patients after surgical treatment for colorectal cancer. Environmental microbiology. 2019;21(2):772-783.

65 Li YD, He KX, Zhu WF. Correlation between invasive microbiota in margin-surrounding mucosa and anastomotic healing in patients with colorectal cancer. World Journal of Gastrointestinal Oncology. 2019;11(9):717-728.

66 Yu SY, Xie YH, Qiu YW, Chen YX, Fang JY. Moderate alteration to gut microbiota brought by colorectal adenoma resection. Journal of Gastroenterology and Hepatology (Australia). 2019;34(10):1758-1765.

67 Wang HF, Li LF, Guo SH, Zeng QY, Ning F, Liu WL, et al. Evaluation of antibody level against Fusobacterium nucleatum in the serological diagnosis of colorectal cancer. Sci Rep. 2016;6:33440.

68 Cole JR, Wang Q, Cardenas E, Fish J, Chai B, Farris RJ, et al. The Ribosomal Database Project: improved alignments and new tools for rRNA analysis. Nucleic Acids Res. 2009;37(Database issue):D141-145.

69 Pruesse E, Quast C, Knittel K, Fuchs BM, Ludwig W, Peplies J, et al. SILVA: a comprehensive online resource for quality checked and aligned ribosomal RNA sequence data compatible with ARB. Nucleic Acids Res. 2007;35(21):7188-7196.

70 McDonald D, Price MN, Goodrich J, Nawrocki EP, DeSantis TZ, Probst A, et al. An improved Greengenes taxonomy with explicit ranks for ecological and evolutionary analyses of bacteria and archaea. Isme j. 2012;6(3):610-618.
